# Long-term cancer risk in historic cohorts of patients with adolescent idiopathic scoliosis: a systematic review

**DOI:** 10.1007/s43390-025-01176-y

**Published:** 2025-09-12

**Authors:** F. D. Højsager, L. W. Laursen, R. Castelein, A. Simony

**Affiliations:** 1 Department of Orthopedic Surgery, The Region of Southern Denmark University Hospital, Sygehusvej 24, 6000 Kolding, Denmark; 2 Department of Orthopedic Surgery, University Medical Center Utrecht, Utrecht, The Netherlands; 3 Department of Regional Health Research, University of Southern Denmark, Odense, Denmark

**Keywords:** Cancer risk, Cancer mortality, Scoliosis, Systematic review, Historical Data

## Abstract

**Purpose:**

To evaluate the long-term cancer risks associated with AIS, focusing on the roles of genetic predispositions and radiation exposure.

**Methods:**

A comprehensive systematic search was conducted on August 5, 2024, across PubMed, EMBASE, Scopus, Cochrane Libraries, and CINAHL, covering studies from 1947 onward. Human studies on patients with scoliosis diagnosed before age 20 were included. For cancer assessment, both risk, incidence and mortality were included. Studies were excluded if they focused solely on congenital or secondary scoliosis. Screening and quality assessment were conducted using Covidence. The first author performed the initial screening, while the first and second authors conducted full-text assessments and quality assessment collaboratively, with an agreement score of 0.83.

**Results:**

Seven studies from the USA, Australia, Denmark and The Netherlands were identified. Notable findings included elevated breast cancer risks among US cohorts, linked to historical radiographic practices that delivered higher radiation doses. None of the included studies assessed genetic etiologies of cancer. Risk of bias in the studies were generally attributed to selection bias and underreporting of characteristics and confounding variables. While most studies included either showed a tendency or a significant association towards an association between scoliosis and risk of cancer, it was mainly based on data before 1990 with exposure to radiation several orders of magnitude larger than modern standards. These changes could be a major factor in the risk of cancer identified in historical cohorts.

**Conclusion:**

This review highlights the importance of continued research, including the effect of modern examination techniques, such as EOS, MRI on rates of cancer in modern populations.

**Supplementary Information:**

The online version contains supplementary material available at 10.1007/s43390-025-01176-y.

## Introduction

Adolescent idiopathic scoliosis (AIS) is the most common form of idiopathic scoliosis with a prevalence of > 1% [1]. Both surgery and conservative measures with bracing are closely monitored through radiographs, with some estimates ranging from 10 to 50 examinations throughout adolescence [2]. The dose of ionizing radiation which is absorbed in the body during X-rays varies depending on the tissue and projection [3]. Radiation dose to the breast of one full spine radiograph has largely reduced throughout the years. Absorbed radiation dose towards the breast has previously been estimated at 0.780 centigray (cGy), if examined between years 1940–1959 in AP projection on children below age 13 [4]. Throughout the years, this is estimated to have been reduced to 0.470 cGy in years 1960–1975 and to 0.125 cGy in 1976–1989. Throughout this period, PA projections are estimated to expose the patients to 0.003 cGy for a full radiograph [4]. As exposure to ionizing radiation is associated with risk of cancer [5–7], previously estimated at an excess relative risk of 5.4/Gy for women with scoliosis [4], it has previously been assessed whether there is an increase in cancer rate among AIS patients [8]. The most predominant focus has been on breast cancer, due to scoliosis often being diagnosed around sensitive time frames of breast development, and the breasts being in the frame of the radiographs.

To our knowledge, no review has focused on cancer risk in scoliosis, although a systematic review has focused on the adverse effects of radiation [9]. Therefore, the aim of this review is to address whether scoliosis is associated with an increase in cancer incidence with either genetic predispositions or exposure to ionizing radiation as possible explanations.

## Methods

### Search strategy

On August 5, 2024, a systematic literature search was conducted using PubMed, EMBASE, Scopus, Cochrane Libraries, and CINAHL. The strategy was designed to obtain every report regarding cancer and scoliosis, which also included either genetic analysis or mentioned radiation exposure. A summary of the strategy is enclosed in Table 1. Search strings for the databases separately are enclosed in Supplementary Tables S1–S5. MeSH terms were included, however, to include literature that has not yet been assigned MeSH terms, searches were made both for full text and MeSH terms where applicable. Publications from 1947 and up until the date of the search were included. For EMBASE, however, the latest update was three days before searching. The search strategy did not use queries for language selection or study design.

**Table 1:** Search strategy

Scoliosis query	Cancer query	Exposure and genes query
Scoliosis	Cancer	Radiation exposure
Idiopathic scoliosis	Neoplasm	Genetics
	Leukemia	Genetic linkage
	Lymphoma	Chromosome mapping
	Malignant melanoma	

Each search term in the query is joined by an OR operator, while the blocks are joined by AND operators

The first author (FH) conducted the search and removed duplicates by use of Covidence [10]. Using the online software platform of Covidence, the abstracts were screened using predetermined inclusion and exclusion criteria.

Inclusion criteria were

- Studies in humans
- Studies involving patients with adolescent idiopathic scoliosis
- Studies where scoliosis was diagnosed prior to age 20 years
- Studies assessing cancer
- Studies with long-time follow-up (> 10 years)

Exclusion criteria were

- Studies only including patients with congenital, neuromuscular, syndromic or secondary scoliosis
- Studies only assessing basal cell carcinoma or cervix uteri cancer

All inclusion and none of the exclusion criteria had to be fulfilled for the article to be included.

Screening of full-text articles was done by the first and last authors (FH & AS).

### Quality assessment

Quality assessment was done using Covidence [10]. Risk of bias was assessed using the STROBE guidelines as a checklist. Two authors (FH & LL) assessed the studies separately and compared results after completion of the assessment. All 22 criteria in the STROBE guidelines were attributed equally in the quality assessment. After initial quality assessment, disagreements were resolved by discussion until agreement had been reached. Furthermore, notable limitations of the studies were noted during the discussions of quality assessment.

## Results

**Fig. 1:**
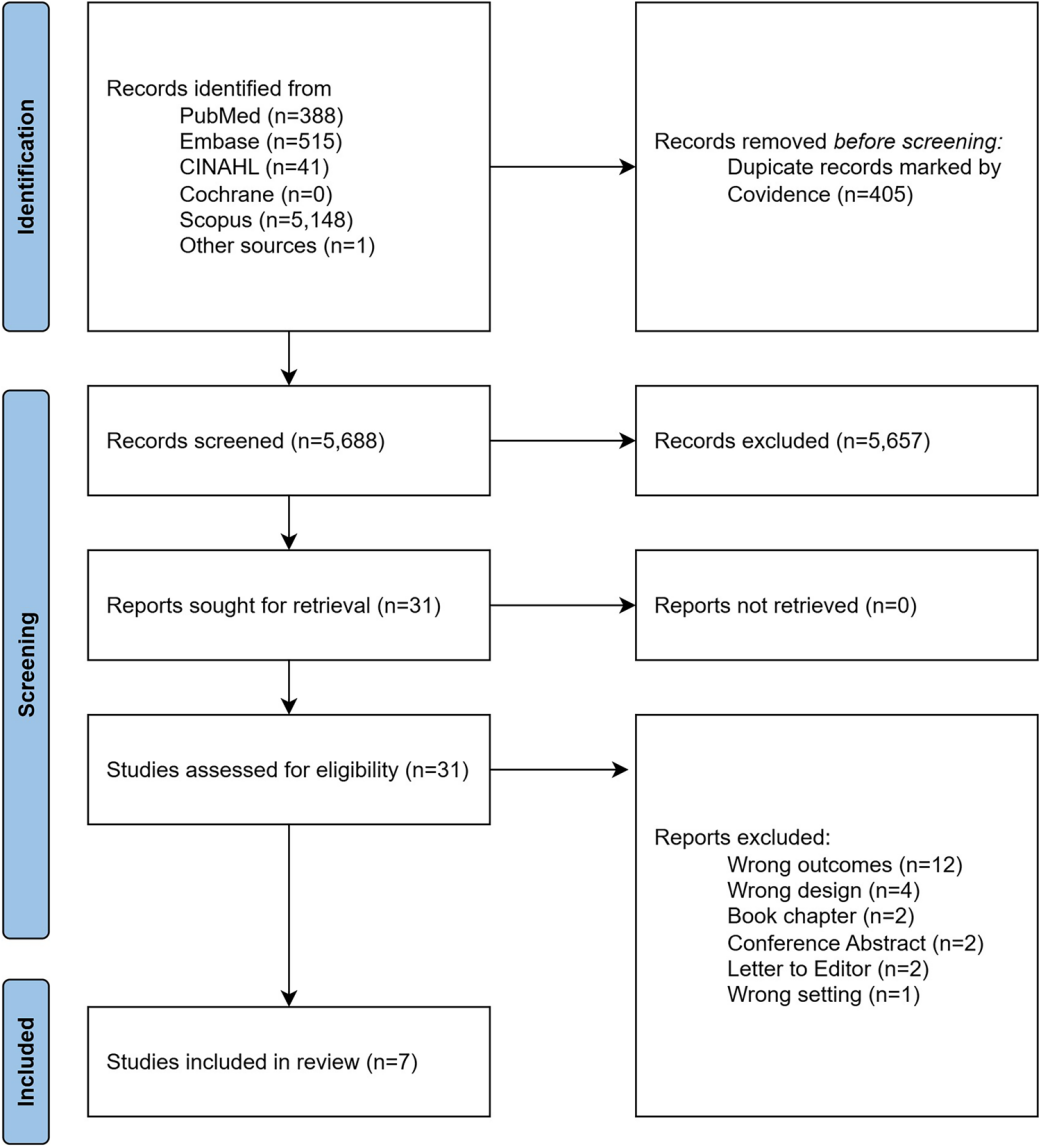
PRISMA flow chart of search strategy

An overview of the search is presented in Fig. 1. The search strategy yielded a total of 6.093 records, of which 5.688 remained after removal of duplicates in Covidence. A vast majority of the records originated from Scopus.

After screening of records, 31 reports remained, all of which were able for retrieval. After full text review, the strategy yielded a total of 7 articles [4, 8, 11–15].

Main characteristics of the articles are presented in Table 2. Most studies identified as retrospective cohort. Although Heijboer et al. [12] described their study as cross-sectional, they were included as their study design was similar to the other included studies. The studies are listed in Table 3 with a summary of the findings across the studies. All included studies addressed radiation to some extent but no studies addressed genetic predisposition.

**Table 2:** Basic characteristics of included studies

Author (year)	Study design	Country	Inclusion period	Sample size	Time of scoliosis diagnosis	Cutoff for cancer diagnosis	Mean age at follow-up	Special characteristics
Cundy et al. [11]	Retrospective cohort	Australia	1979 – Jun 2011	865	Before age 19	December 31 2015	N/A	Only patients who underwent instrumented spinal surgery
Doody et al. [4]	Retrospective cohort	USA	1912–1965	5466	Before age 20	January 1 1997	51 years (range 2–89)	Originating from US Scoliosis Cohort [4]
Heijboer et al. [12]	Retrospective cohort^a^	Netherlands	Jan 1981–Jan 1995	337	Before age 19	N/A	44 years (SD 6.6)	Only idiopathic scoliosis (juvenile and adolescent)
Hoffmann et al. [13]	Retrospective cohort	USA	1935–1965	973	Before age 20	1983–1986	41.4 years	Diagnosis of scoliosis or kyphosis
Ronckers et al. [14]	Retrospective cohort	USA	1912–1965	3010	Before age 20	1992	51.3 years	Originating from US Scoliosis Cohort [4]
Ronckers et al. [15]	Retrospective cohort	USA	1912–1965	5513	Before age 20	December 31, 2004	58.3 years	Originating from US Scoliosis Cohort [4]
Simony et al. [8]	Retrospective cohort	Denmark	1983–1990	211	Before age 19	N/A	37.6 years	Only AIS patients

Each search term in the query is joined by an OR operator, while the blocks are joined by AND operators

^a^Described by the authors as cross-sectional, however, due to the similarities to a retrospective cohort study, they are categorized as such

**Table 3:** Findings of the studies included

Author (year)	Main findings	Other notable findings	Notable limitations
Cundy et al. [11]	SIR (95% CI), all types Total: 1.00 (0.50–1.79) Female: 0.83 (0.33–1.70) Male: 1.33 (0.36–3.40)		Male SIR based on 4 cases No reported follow-up time Avg. person-years contribution 18.4 years
Doody et al. [4]	SMR (95% CI), breast Female: 1.96 (1.3–2.1)	No excess mortality for lung cancer Increased SMR for breast cancer with number of spinal surgeries Increased SMR for breast cancer with number of radiographic exams	Most examinations prior to 1976, yielding much larger radiation dose Almost exclusively using anteroposterior view Both factors contributing to much larger radiation dose to breast tissue, than what is expected presently
Heijboer et al. [12]	SPR (95% CI), all non-BCC Female: 1.10 (N/D) (*p* = 0.43)	No significant difference between cancer prevalence in patients treated surgically compared to non-surgically (*p* = 0.10) No correlation between total number of radiographs and patients who developed cancer (*p* = 0.90)	Underpowered according to themselves Selection bias, as patients are less likely to smoke relatively to the background population (8.6 v 26%)
Hoffmann et al. [13]	SIR (90% CI), breast Female: 1.8 (1.0–3.0)	Nulliparous women had higher SIR of breast cancer compared to parous women. In both groups SIR increased with radiation dose Apparently lower SIR in women with first X-ray at age 10–14 compared to other age groups	Historical data meaning X-ray exposure was prior to using posteroanterior position, breast shields and other radiation-reducing actions
Ronckers et al. [14]	ERR/Gy (95% CI), breast Female: 2.86 (− 0.07 to 8.62)	Only an increase in ERR in women with a family history of breast cancer	
Ronckers et al. [15]	SMR (95% CI), breast Female: 1.68 (1.38–2.02)	SMR, all cause (95% CI) of 1.46 (1.39–1.54) ERR/Gy of 3.9 for breast cancer	Akin to other studies from the same cohort [4, 13, 14], the present external validity is limited due to radiation being vastly decreased over the past century
Simony et al. [8]	RR (95% CI), all All: 4.8 (2.3–5.8)	Patients who developed endometrial cancer had low BMI and were described as skeletally immature	Small sample and few cases of cancer (*n* = 9) No clearly reported follow-up time

*SIR* Standardized Incidence Ratio, *SMR* Standardized Mortality Ratio, *BCC* basal cell carcinoma, *ERR* excess risk ratio, *Gy* gray

The settings of the studies included in the review were Australia [11], Denmark [8], Netherlands [12] and the United States [4, 13–15], with the four American studies including the same cohort or sub-segments of that cohort.

The included studies had a wide array of outcomes. Two studies [11, 13] assessed the Standardized Incidence Ratio (SIR) of breast cancer. Two studies [4, 15] assessed the Standardized Mortality Ratio (SMR), although both studies included patients from the same cohort. The remaining assessed Standardized Prevalence Ratio (SPR) [12], Excess Risk Ratio per Grey (ERR/Gy) [14], and Risk Ratio (RR) [8]. The American studies had their main focus on breast cancer, while the remaining focused on all cancers. The Dutch study did not include Basal Cell Carcinoma, as it is not registered in the Dutch Cancer Register [12].

As a majority of the included studies were overlapping in their setting, and the outcomes varied, no meta-analysis was performed.

Post-hoc analysis of agreement between authors FH and LL was performed yielding an overall agreement of 0.83 (Table 4).

**Table 4:** Risk of bias assessment of included studies

	Cundy et al. [11]	Doody et al. [4]	Heijboer et al. [12]	Hoffmann et al. [13]	Ronckers et al. [14]	Ronckers et al. [15]	Simony et al. [8]
Does the title or abstract indicate what was done and what was found?	Yes	Yes	Yes	Yes	Yes	Yes	Yes
Does the introduction explain the scientific background?	Yes	Yes	Yes	Yes	Yes	Yes	Yes
Does the introduction state specific objectives?	Yes	Yes	Yes	Yes	Yes	Yes	Yes
Does the method present key elements of the study design?	Yes	Yes	Yes	Yes	Yes	Yes	Yes
Does the method describe the setting, locations, and relevant dates?	Yes	Yes	Yes	Yes	Yes	Yes	No
Does the method give the eligibility criteria, and the sources and methods of selection of participants?	Yes	Yes	Yes	Yes	Yes	Yes	Unclear
Does the method define all outcomes, exposures, predictors, potential confounders?	Yes	Yes	Yes	Yes	Yes	Yes	Yes
Does the method give sources of data and details of methods of assessment?	Yes	Yes	Yes	Yes	Yes	Yes	Yes
Does the method describe any efforts to address potential sources of bias?	Yes	Yes	Yes	Yes	Yes	Yes	Yes
Does the method explain how the study size was arrived at?	Yes	Yes	Yes	Yes	Yes	Yes	No
Does the method explain how quantitative variables were handled in the analyses?	Yes	Yes	Yes	Yes	Yes	Yes	Yes
Does the method describe all statistical methods, describe methods and explain how missing data were addressed?	Yes	Yes	Yes	Yes	Yes	Yes	Yes
Does the results report numbers of participants at all stages and give reasons for non-participation?	Yes	Yes	Yes	Yes	Yes	Yes	Yes
Does the results give characteristics of study participants?	Yes	Yes	Yes	Yes	Yes	Yes	No
Does the results report numbers of outcome events?	Yes	Yes	Yes	Yes	Yes	Yes	Yes
Does the results report unadjusted estimates and their precision?	Yes	Yes	Yes	Yes	Yes	Yes	Yes
Does the results report other analyses done?	No	Yes	Yes	Yes	Yes	Yes	Unclear
Does the discussion summarize key results?	Yes	Yes	Yes	Yes	Yes	Yes	Yes
Does the discussion discuss limitations of the study?	Yes	Yes	Yes	Yes	Yes	Yes	Yes
Does the discussion give a cautious overall interpretation of the results?	Yes	Yes	Yes	Yes	Yes	No	Yes
Does the discussion discuss the external validity of the study?	No	Yes	Yes	Yes	Yes	Yes	Yes
Does the study give the source of funding and the role of funders?	Yes	Yes	Yes	Yes	Yes	Yes	No
Overall risk of bias	Low/moderate	Low	Low	Low	Low	Low	Moderate

Questions are based on STROBE criteriae

### Characteristics

All four American studies were based on the US Scoliosis Cohort, although Hoffmann et al. [13] formally do not describe the US Scoliosis Cohort Study, the study by Doody et al. [4] acknowledges the cohort from Hoffman as a “pilot study”. The US Scoliosis Cohort Study is a historical cohort of women who were diagnosed with scoliosis, kyphosis, lordosis, or kyphoscoliosis in one of 14 medical centers during 1912–1965. Outcomes in the American cohorts were assessed in 1983–1986 [13], 1992 [14], 1997 [4], and 2004 [15]. Mean age at follow-up ranged from 41.4 to 58.3 years between the studies. A majority of the patients included were, due to the time period they were diagnosed, exposed to larger quantities of radiation than modern cohorts. Furthermore, according to Doody et al., almost all radiographs were done using the anteroposterior view, yielding a much larger absorbed radiation dose in the anterior thorax, in turn yielding a larger radiation dose in the breast tissue [4].

Cundy et al. [11] included all patients undergoing instrumented spinal surgery for correction of spinal deformity. Their inclusion period was from 1979 to 2011. They assessed the development of cancer by December 31, 2015 using the South Australian Cancer Registry (SACR). The mean age at follow-up was not enclosed, however, the average follow-up was 18 years (range 0–36 years) and the average age at surgery was 13.9 years, rendering the cohort relatively young. Radiographs were not assessed directly in the cohort. The study furthermore focused on circulating ions from the implants. Due to their timeframe stainless steel implants were mainly used, with titanium only being used in the majority of surgeries from 2003 and onward. They included all causes of scoliosis, with an apparent majority of neuromuscular scoliosis among patients with cancer (63.6%, Table 1), in sharp contrast to the majority of the total cohort being idiopathic (67.4%, Table 1). There were, however, discrepancies between Tables 1 and 2 regarding etiology of the patients’ scoliosis. According to Table 1, 7 of the cancer cases were diagnosed with neuromuscular scoliosis. In Table 2, only 5 cancer cases had neuromuscular scoliosis. Upon contacting the authors, it was revealed, that Table 1 was correct.

Heijboer et al. [12] looked at data from 337 patients treated at OLVG hospital, Amsterdam from January 1981 to January 1995. The cohort solely consisted of patients with idiopathic scoliosis. Follow-up was done by using the Dutch national cancer registry, IKNL. The average age at evaluation was 44 years. By use of logbooks and radiographic records, they assessed both number and type of radiographs, including projection of X-rays. In their study period, the PA view was used almost exclusively.

Lastly, Simony et al. [8] described 211 patients with adolescent idiopathic scoliosis treated between 1983 and 1990 in Denmark. They assessed whether the patients had cancer during their examinations, and by a questionnaire asking specifically about previous cancer diagnosis/treatments. Their mean age at follow-up was relatively low, for the development of cancer, at 37.6 years. They were the only cohort consisting solely of patients with adolescent idiopathic scoliosis, in contrast to Heijboer et al., which consisted of all types of idiopathic scoliosis.

### Quality assessment

The American studies were based on a large national cohort with clearly stated inclusion criteria. Roughly 40% of the included patients were treated at a Shriners Hospital, which during the inclusion period offered treatment free of charge. This may yield a small selection bias. Furthermore, due to the long follow-up period and use of questionnaires and telephone tracers, the studies were prone to recall bias. The recall bias was especially significant in relation to family history of breast cancer, as knowledge of family health matters is more likely when a person has the same disease. Overall the studies were of high quality albeit with historical data, which may diminish the external validity (Table 4).

Cundy et al. clearly stated their inclusion criteria. With an apparent inclusion of all Australian patients, which had verifiable implants and did not have surgery prior to their inclusion period, or cancer prior to surgery, the risk of selection bias appeared negligible. They did, however, not account for the mean age at follow-up, nor did they factor in death as a competing risk for developing cancer. Furthermore, the cancer cases were predominant in the group of neuromuscular scoliosis patients. With a total of 11 cases, of which the data were stratified by gender, the study was most likely underpowered. The authors did, however, not assess this in their discussion. Due to these factors, authors FH and LL agreed on a low/moderate risk of bias in the study (Table 4).

Heijboer et al. presented a high-quality study, which clearly stated the inclusion criteria. With a majority of their study population apparently having AIS, it was highly relevant for this review. It did, however, have some shortcomings, with the major one being an apparent selection bias, where study participants were less likely to smoke than patients without scoliosis. Furthermore, according to the authors, their study was underpowered.

Although the Simony et al. study had the patient group most relevant for this review, the study had some notable risk of bias. No apparent description of the setting, nor the reasoning for the number of included patients were given. The eligibility criteria were unclear in the method, with included patients being described as “eligible consecutive”. Very few baseline characteristics were presented for the patient population. Lastly, no source of funding was disclosed. All in all, the two authors FH and LL deemed the total risk of bias “moderate” based on these observations﻿ (Table 4).

### Study outcomes

#### Incidence

Cundy et al. and Hoffmann et al. reported Standardized Incidence Ratios (SIR), while Heijboer et al. reported Standardized Prevalence Ratio (SPR). Cundy et al. found a total SIR of 1.00 in their group, which when stratified on gender revealed an apparent discrepancy between genders, with male SIR > 1 and female SIR < 1. Both were, however, non-significant and were by the authors attributed to a small number of cases (for males 4 observed cases compared to 3 expected). Furthermore, as they included all types of cancer only one case was breast cancer, with others including leukemia and several types of carcinomas. Hoffman et al. found 11 cases of breast cancer compared to 6 expected among the 1030 included women. This yielded an SIR of 1.82, which was not significant, as their 90% CI included 1.0. They did not report other types of cancer.

Heijboer et al. calculated a SPR, which corresponds to the SIR, but using a prevalence as a comparator among the groups. They found an SPR of 1.10, which was not significant. This was based on a prevalence of 5% of all non-BCC cancers breast cancer accounted for 23.5% of the included cancers.

#### Mortality

Doody et al. and Ronckers et al. reported the Standardized Mortality Ratio (SMR). Both had a main focus on breast cancer, but also reported mortality for other cancer types. Furthermore, both were based on the American Scoliosis Cohort. Doody et al. found a significant increase in mortality from breast cancer of 1.96 (1.3–2.1), similarly Ronckers et al. found an SMR of 1.86 (1.38–2.02) of breast cancer.

### Risk of development

Ronckers et al. and Simony et al. reported alterations in risk of cancer development, with Ronckers et al. focusing on a cause-effect relationship of radiation, while Simony et al. assessed differences in risk of cancer between patients with and without AIS. Ronckers et al. assumed a linear no threshold relationship and assessed the excess risk ratio per gray of absorbed radiation dose. They found an ERR/Gy of 2.86, which although not significant (*p* = 0.058), was rather robust in their sensitivity analysis. Furthermore, they identified that the effect was attenuated when there was no family history of breast cancer.

Simony et al. found relative risk of 4.8 (95% CI 2.3–5.8) for any cancer, in AIS patients. They furthermore found a tendency of patients with endometrial cancer having a lower BMI and being skeletally immature by the time of radiographic examination.

## Discussion

### General interpretation

This review found an apparent increase in mortality and incidence of cancer in women with scoliosis. The included studies predominantly assessed breast cancer. Most included studies, were however historical when compared to modern radiography standards. Regrettably no included studies addressed genetic predisposition for cancer, or any genetic risk factors.

### Limitation of evidence

The included studies had several notable limitations. While the American studies of Hoffman, Doody, and Ronckers et al. were of a high quality, their external validity is limited due to two notable factors. The standard projection for X-rays of the spine has changed to a PA view, rather than an AP view, and due to advances in technology, such as introduction of digital radiography, patients are exposed to less ionizing radiation in modern assessment of scoliosis. The change in projection yields a change in the pattern of which tissue absorbs X-rays. With a PA projection, X-rays will be blocked by ribs, lungs and the spine before being absorbed in breast tissue. As their main findings are based on a hypothesis of breast tissue being sensitive towards radiation, the change in projection should be considered a notable risk-mitigating factor. In their reporting of exposure to radiation, they reported absorbed dose in cGy rather than effective dose in Sievert (Sv). As such, their reported outcome does not reflect the stochastic nature of radiation absorption. This should be considered, as a direct conversion from cGy to cSv should also take into account the weighting factor of e.g. 0.12, to accommodate the difference between absorbed dose and effective dose to the breast. Furthermore, the technological advances in X-ray procedures, such as slot scanning or digital radiography, has yielded a reduction in exposure by several orders of magnitude since the inclusion of patients in the American studies [16–18]. On the contrary, increased use of CT navigation has also altered preoperative and intraoperative exposure to radiation [19]. In a former study, low-dose O-arm imaging resulted in an average radiation dose of 1.11 mSv, compared with 0.27 mSv for C-arm fluoroscopy, representing about a fourfold difference in pedicle screw placement [20]. In another study, low-dose preoperative CT was reported at effective doses as low as 0.7 mSv [21]. This change in radiation exposure pattern could largely affect the risk of radiation-induced cancers, as addressed by Ronckers et al. Furthermore, the emerging role of AI-assisted MRI in operative planning could further diminish the role of radiation in scoliosis surgeries [22]. Although Ronckers et al. assume a no-threshold linear model for excess risk per Gy, they only assessed whether it was linear at extreme levels of radiation and it is uncertain whether linearity is still present in lower doses.

Although the remaining studies had inclusion periods where PA projections were more likely to be used, they suffered from being underpowered. Heijboer et al. included this in their own discussion, as they had a sample size of 337 patients. Cundy et al. did not discuss this, but based their findings on 11 cases of cancer among their sample size of 865 patients. Simony had a small sample size of 211 patients and a total of 9 cancer cases. Heijboer et al. raised the question of their own validity in relation to selection bias. Their included cohort was less likely to smoke compared to the background population, which might limit the validity of their findings. Simony et al. included a few characteristics of their patients, which limits the knowledge of whether these were representative of a general scoliosis population. Both Cundy and Simony did not clearly reported a follow-up period or time frame, which makes comparison across studies difficult.

### Limitation of review

This review has notable limitations. The review relies on some of the largest databases, but preprints were omitted. Included preprints could, in principle, have included more literature in the review. However, due to preprints not being peer reviewed, concerns have been raised about the quality of preprint research [23], we, therefore, only included peer-reviewed articles.

Secondly, even though Covidence filtered a large amount of duplicates, further manual identification was done during abstract screening, where a large amount of duplicates were identified. These were simply marked as ineligible for full-text retrieval. This might be due to the fact that EMBASE and MEDLINE have a large overlap in included journals. Further, of the articles in the included databases, articles originating from SCOPUS accounted for 84.5%. As the search strategy partly relied on abbreviations, and the included journals in SCOPUS cover a wide array of non-medical fields, a vast quantity of these did not meet the inclusion criteria.

Although the scope of the review was risk of cancer, with no restrictions on type of cancer, included studies mainly focused on breast cancers, which limits the implications to these diseases.

Furthermore, we chose to exclude papers if they only assessed basal cell carcinoma or cervix uteri cancer. Although this might have reduced the pool of available papers, these cancers are registered and considered as dysplasias and not cancers in some registers like NORDCAN or IKNL [8, 12].

Regrettably, no studies were identified that addressed the role of genetics in cancer risk, which led to this review only fulfilling its aim partially.

Lastly, the heterogeneity of outcomes in the included literature, as well as the homogeneity of the included population in 4 of 7 studies, hindered meta-analysis, yielding a narrative nature of this review. Several studies were furthermore underpowered, which limits the implications.

### Implications of research

The general implication of the review is that there might be an increased in risk of cancer among patients with scoliosis. Notably, published material has focused largely on breast cancer. Most included studies were based on historical data, which can be seen as a consequence of the follow-up needed to ensure a mean age relevant to the development of cancer. Curiously, several studies, even modern ones, had a low mean age of cancer debut. However, due to notable limitations, mostly regarding the magnitude of radiation exposure, replications of the studies are needed in modern settings e.g. with inclusion of patients within a time frame in which use of PA projections can be reasonably assumed. Such replications could also focus on the role of repeated exposure towards radiation, as the cumulative effective dose was not addressed in the included studies. Such studies could help clarify whether the findings from historical cohorts still apply to patients today. Furthermore, as the radiation exposure in diagnostics has decreased, this yields a larger relative radiation load from intra-operative radiation, which could be accounted for in future studies. Due to these changes, a study like this would most likely only be able to assess early onset cancer, as patients born in periods of time that used the PA projection in diagnosis are still relatively young. It could still be relevant to test, as the mean age of patients in the included studies was relatively low. Focus of such studies should stretch beyond breast cancer, as these are underreported in the current literature. The knowledge gap stretches further, as no studies addressing genetic predispositions were identified. This could be relevant as linkage of scoliosis to e.g. VANGL1 [24, 25], which is also linked to breast cancer [26] is discussed.

## Conclusion

Although few studies implicate an increased risk and mortality of cancer, predominantly breast cancer, this is primarily based on historical cohorts. This review also highlights the need of large-scale modern studies to address cancer risk in scoliotic women exposed to lower doses of radiation.

## Supplementary Information

Below is the link to the electronic supplementary material.

Supplementary file1 (DOCX 18 kb)

## References

[1] Lonstein JE, Bjorklund S, Wanninger MH, Nelson RP (1982) Voluntary school screening for scoliosis in Minnesota. J Bone Jt Surg Am 64(4):481–488 6802853

[2] Oakley PA, Ehsani NN, Harrison DE (2019) The scoliosis quandary: are radiation exposures from repeated X-rays harmful? Dose-Response 17(2):1559325819852810 31217755 10.1177/1559325819852810 PMC6560808

[3] Tafti D, Maani CV (2023) X-ray production. StatPearls. StatPearls Publishing, Treasure Island 30725731

[4] Doody MM, Lonstein JE, Stovall M, Hacker DG, Luckyanov N, Land CE et al (2000) Breast cancer mortality after diagnostic radiography: findings from the US Scoliosis Cohort Study. Spine 25(16):2052–2063 10954636 10.1097/00007632-200008150-00009

[5] UNSCEAR (2000) UNSCEAR 2000: the united nations scientific committee on the effects of atomic radiation. Health Phys 79(3):314 10949259

[6] Land CE, Boice JD Jr, Shore RE, Norman JE, Tokunaga M (1980) Breast cancer risk from low-dose exposures to ionizing radiation: results of parallel analysis of three exposed populations of women. J Natl Cancer Inst 65(2):353–376 6931253

[7] Boice JD Jr, Land CE, Shore RE, Norman JE, Tokunaga M (1979) Risk of breast cancer following low-dose radiation exposure. Radiology 131(3):589–597 441361 10.1148/131.3.589

[8] Simony A, Hansen EJ, Christensen SB, Carreon LY, Andersen MO (2016) Incidence of cancer in adolescent idiopathic scoliosis patients treated 25 years previously. Eur Spine J 25:3366–3370 27592106 10.1007/s00586-016-4747-2

[9] Luan F-J, Wan Y, Mak K-C, Ma C-J, Wang H-Q (2020) Cancer and mortality risks of patients with scoliosis from radiation exposure: a systematic review and meta-analysis. Eur Spine J 29(12):3123–3134 32852591 10.1007/s00586-020-06573-7

[10] software Csr (2024) www.covidence.org: Veritas Health Innovation, Melbourne, Australia

[11] Cundy PJ, Venugopal K, Antoniou G, Brooks F, Freeman BJ, D’Onise K (2020) Do children with spinal deformity who have metal implants and frequent exposure to X-rays increase their risk of cancer? Spine 45(17):1200–1207 32355145 10.1097/BRS.0000000000003507

[12] Heijboer RR, Heemskerk JL, Vorrink SN, Kempen DH (2024) The prevalence of cancer in Dutch female patients with idiopathic scoliosis compared with the general population. J Clin Med 13(9):2616 38731145 10.3390/jcm13092616 PMC11084711

[13] Hoffman DA, Lonstein JE, Morin MM, Visscher W, Harris BS III, Boice JD Jr (1989) Breast cancer in women with scoliosis exposed to multiple diagnostic X rays. JNCI J Natl Cancer Inst 81(17):1307–1312 2769783 10.1093/jnci/81.17.1307

[14] Ronckers CM, Doody MM, Lonstein JE, Stovall M, Land CE (2008) Multiple diagnostic X-rays for spine deformities and risk of breast cancer. Cancer Epidemiol Biomark Prev 17(3):605–613 10.1158/1055-9965.EPI-07-2628 18349278

[15] Ronckers CM, Land CE, Miller JS, Stovall M, Lonstein JE, Doody MM (2010) Cancer mortality among women frequently exposed to radiographic examinations for spinal disorders. Radiat Res 174(1):83–90 20681802 10.1667/RR2022.1 PMC3982592

[16] Cool J, Streekstra G, Van Schuppen J, Stadhouder A, Van den Noort J, Van Royen B (2023) Estimated cumulative radiation exposure in patients treated for adolescent idiopathic scoliosis. Eur Spine J 32(5):1777–1786 36943485 10.1007/s00586-023-07651-2

[17] Luckner C, Weber T, Herbst M, Ritschl L, Kappler S, Maier A (2021) A phantom study on dose efficiency for orthopedic applications: comparing slot-scanning radiography using ultra-small-angle tomosynthesis to conventional radiography. Med Phys 48(5):2170–2184 33368397 10.1002/mp.14680

[18] Luo TD, Stans AA, Schueler BA, Larson AN (2015) Cumulative radiation exposure with EOS imaging compared with standard spine radiographs. Spine Deform 3(2):144–150 27927305 10.1016/j.jspd.2014.09.049

[19] Striano BM, Crawford AM, Verhofste BP, Hresko AM, Hedequist DJ, Schoenfeld AJ et al (2024) Intraoperative navigation increases the projected lifetime cancer risk in patients undergoing surgery for adolescent idiopathic scoliosis. Spine J 24(6):1087–1094 38262498 10.1016/j.spinee.2024.01.007

[20] Su AW, McIntosh AL, Schueler BA, Milbrandt TA, Winkler JA, Stans AA et al (2017) How does patient radiation exposure compare with low-dose O-arm versus fluoroscopy for pedicle screw placement in idiopathic scoliosis? J Pediatr Orthop 37(3):171–177 27453221 10.1097/BPO.0000000000000608

[21] Sullivan MH, Yu L, Schueler BA, Nassr A, Guerin J, Milbrandt TA et al (2024) Radiation exposure in navigated techniques for AIS: is there a difference between pre-operative CT and intraoperative CT? Spine Deform 12(2):349–356 37870680 10.1007/s43390-023-00772-0

[22] Lafranca PP, Rommelspacher Y, Walter SG, Muijs SP, van der Velden TA, Shcherbakova YM et al (2025) The safety and accuracy of radiation-free spinal navigation using a short, scoliosis-specific BoneMRI-protocol, compared to CT. Eur Spine J (2025). 10.1007/s00586-025-0915 10.1007/s00586-025-09151-x PMC12858448 40691585

[23] Malički M, Jerončić A, Ter Riet G, Bouter LM, Ioannidis JP, Goodman SN et al (2020) Preprint servers’ policies, submission requirements, and transparency in reporting and research integrity recommendations. JAMA 324(18):1901–1903 33170231 10.1001/jama.2020.17195 PMC7656281

[24] Andersen MR, Farooq M, Koefoed K, Kjaer KW, Simony A, Christensen ST et al (2017) Mutation of the planar cell polarity gene VANGL1 in adolescent idiopathic scoliosis. Spine 42(12):E702–E707 27755493 10.1097/BRS.0000000000001927

[25] Xu L, Sheng F, Xia C, Feng Z, Qiu Y, Zhu Z (2018) VANGL1 is not associated with the susceptibility of adolescent idiopathic scoliosis in the Chinese population. Spine 43(10):E580–E584 29189642 10.1097/BRS.0000000000002497

[26] Hatakeyama J, Wald JH, Printsev I, Ho H-YH, Carraway KL (2014) Vangl1 and Vangl2: planar cell polarity components with a developing role in cancer. Endocr Relat Cancer 21(5):R345–R356 24981109 10.1530/ERC-14-0141 PMC4332879

