# Supplementary material for: Long-term cancer risk in historic cohorts of patients with adolescent idiopathic scoliosis: a systematic review
Source: Spine Deform. 2025 Sep 12;14(1):93–102. doi: 10.1007/s43390-025-01176-y (PMC12816099; doi:10.1007/s43390-025-01176-y)
Supplement: Supplementary file 1 — Supplementary file1 (DOCX 18 kb) [file 43390_2025_1176_MOESM1_ESM.docx]

Table S1 - Results from PubMed search performed on August 5, 2024.

| Query | Results |
| --- | --- |
| Scoliosis (All fields) | 32,197 |
| Scoliosis (MeSH) | 18,276 |
| Idiopathic scoliosis (All fields) | 10,124 |
| 1 OR 2 OR 3 | 32,197 |
|  |  |
| Cancer (All fields) | 5,154,255 |
| Neoplasms (MeSH) | 3,559,087 |
| Leukemia (All fields) | 379,552 |
| Lymphoma (All fields) | 322,917 |
| Malignant Melanoma (All fields) | 165,819 |
| 5 OR 6 OR 7 OR 8 OR 9 | 5,252,562 |
|  |  |
| Radiation Exposure (All fields) | 166,614 |
| Radiation Exposure (MeSH) | 60,676 |
| Genetics (All fields) | 5,167,947 |
| Genetics (MeSH) | 91,100 |
| Chromosome Mapping (All fields) | 144,282 |
| 11 OR 12 OR 13 OR 14 OR 15 | 5,318,732 |
|  |  |
| 4 AND 10 AND 16 | 388 |

Table S2 – Results of EMBASE search performed on August 5, 2024. Note that the last update of EMBASE was August 2, according to EMBASE.

| Query | Results |
| --- | --- |
| exp Scoliosis/ | 48,001 |
| exp Idiopathic scoliosis/ | 10,535 |
| 1 OR 2 | 48,001 |
|  |  |
| exp cancer/ | 4,76,659 |
| exp neoplasm/ | 6,275,945 |
| exp leukemia/ | 423,804 |
| exp lymphoma/ | 372,146 |
| exp malignant melanoma/ | 219,118 |
| 4 OR 5 OR 6 OR 7 OR 8 | 6,275,945 |
|  |  |
| exp radiation exposure/ | 292,835 |
| exp genetics/ | 1,450,270 |
| exp chromosome mapping/ | 4,781 |
| 10 OR 11 OR 12 | 1,733,185 |
|  |  |
| 3 AND 9 AND 13 | 515 |

Table S3 - Results from Scopus search performed on August 5, 2024.

| Query | Results |
| --- | --- |
| ALL(scoliosis) | 87,868 |
| ALL(”idiopathic scoliosis”) | 32,665 |
| 1 or 2 | 87,868 |
|  |  |
| ALL(cancer) | 9,240,597 |
| ALL(neoplasm) | 3,418,438 |
| ALL(leukemia) | 1,499,563 |
| ALL(lymphoma) | 992,123 |
| ALL(”malignant melanoma”) | 187,530 |
| 4 or 5 or 6 or 7 or 8 | 10,346,410 |
|  |  |
| ALL(”radiation exposure”) | 225,357 |
| ALL(genetics) | 6,542,486 |
| ALL(”chromosome mapping”) | 86,573 |
| 10 or 11 or 12 | 6,743,459 |
|  |  |
| 3 and 9 and 13 | 5,168 |

Table S4 - Results from Cochrane Libraries search performed on August 5, 2024.

| Query | Results |
| --- | --- |
| MeSH descriptor: [Scoliosis] explode all trees | 754 |
| scoliosis, ti,ab,kw | 1,905 |
| idiopathic scoliosis, ti,ab,kw | 1,006 |
| #1 or #2 or #3 | 1,905 |
|  |  |
| cancer, ti,ab,kw | 236,332 |
| MeSH descriptor: [Neoplasms] explode all trees | 125,865 |
| leukemia, ti,ab,kw | 18,399 |
| lymphoma, ti,ab,kw | 15,351 |
| malignant melanoma, ti,ab,kw | 1,449 |
| #6 or #7 or #8 or #9 | 143,050 |
|  |  |
| radiation exposure, ti,ab,kw | 4,107 |
| MeSH descriptor: [Radiation Exposure] explode all trees | 2,300 |
| genetics, ti,ab,kw | 30,826 |
| MeSH descriptor: [Genetics] in all MeSH products | 1,756 |
| chromosome mapping, ti,ab,kw | 123 |
| #11 or #12 or #13 or #14 or #15 | 37,278 |
|  |  |
| #4 and #10 and #16 | 0 |

Table S5 - Results from CINAHL search performed on August 5, 2024.

| Query | Results |
| --- | --- |
| Scoliosis | 10,822 |
| Idiopathic scoliosis | 4,291 |
| S1 OR S2 | 10,822 |
|  |  |
| cancer | 564,042 |
| neoplasm | 666,896 |
| leukemia | 36,976 |
| lymphoma | 37,154 |
| malignant melanoma | 13,290 |
| S4 OR S5 OR S6 OR S7 OR S8 | 878,198 |
|  |  |
| Radiation exposure | 17,511 |
| genetics | 189,257 |
| chromosome mapping | 1,494 |
| S10 OR S11 OR S12 | 206,767 |
|  |  |
| S3 AND S9 AND S13 | 41 |
